# Influence of Culture Media and Fermentation Conditions on Growth, Sporulation, and Biomass Production of *Fusarium venenatum* CML3311

**DOI:** 10.3390/microorganisms14081709

**Published:** 2026-08-04

**Authors:** Carine dos Reis Teixeira, Katharine Valéria Saraiva Hodel, Lavinia Laís Nepomuceno da Silva, Leticia Alencar Pereira Rodrigues, Marcelo Andrés Umsza-Guez

**Affiliations:** 1 Food Science Postgraduate Program, Faculty of Pharmacy, Federal University of Bahia, Salvador 40170-100, BA, Brazil; carine.teixeira@ufba.br; 2 SENAI Institute for Innovation in Advanced Health Systems, SENAI CIMATEC University, Salvador 41650-010, BA, Brazil; katharine.hodel@fieb.org.br (K.V.S.H.); leticiap@fieb.org.br (L.A.P.R.); 3 Department of Biotechnology, Institute of Health Sciences, Federal University of Bahia (UFBA), Salvador 40231-300, BA, Brazil; lavinianepomuceno@ufba.br

**Keywords:** submerged fermentation, fungal biomass, alternative proteins

## Abstract

The increasing demand for alternative protein sources has stimulated interest in fungal biomass as a food ingredient. Although industrial strains of *Fusarium venenatum* have been extensively investigated, strain-specific information on conidial production, biomass accumulation, and crude protein content remains limited for strains maintained in microbial culture collections. This study evaluated the growth and sporulation of *F. venenatum* CML3311 in different solid media and investigated biomass and crude protein production under submerged fermentation conditions. Growth and sporulation were assessed on Yeast Malt Agar (YMA), Dichloran Rose Bengal Chloramphenicol Agar (DRBC), Potato Dextrose Agar (PDA), and Sabouraud Dextrose Agar (SDA). Submerged fermentations were conducted using a 3^2^ factorial design evaluating temperature (26–30 °C) and agitation (160–200 rpm) over 72 h. Biomass production was determined gravimetrically, and crude protein content was quantified by the Kjeldahl method. DRBC promoted the highest sporulation, reaching 4.63 × 10^6^ conidia/mL after 28 days. Biomass production increased during the first 48 h of cultivation, with the highest yields observed at 28–30 °C (6.4–6.5 g/L). Crude protein content ranged from 25.45% to 56.12% (dry basis), with the highest value obtained after 24 h at 28 °C and 200 rpm. These findings provide a strain-specific basis for inoculum preparation and submerged cultivation of *F. venenatum* CML3311 and identify conditions to be further investigated in process-optimization and safety-assessment studies.

## 1. Introduction

The increasing demand for alternative protein sources is a well-established concern in the literature and has guided extensive research on this topic. Projections indicating that the world population may reach 9.7 billion by 2050 have intensified the debate regarding the capacity of current livestock production systems to meet future protein requirements, while also reinforcing concerns about associated environmental impacts [1,2,3,4,5,6,7,8,9,10,11,12,13,14,15,16,17,18]. In this context, the search for new protein sources has focused on alternatives capable of meeting this growing demand. Alternative protein sources to meat include those derived from plants, insects, and microorganisms [19]. Among these, mycoprotein, a fungal-derived biomass, has gained attention in several studies due to its nutritional profile [20,21,22].

The production of fungal biomass for food applications depends directly on the microorganism’s response to the cultivation conditions employed. In filamentous fungi, medium composition, nutrient availability, temperature, and oxygen transfer influence not only mycelial growth, but also sporulation, morphology, and the efficiency of substrate conversion into biomass [23,24,25]. Therefore, defining suitable cultivation conditions represents an important step in the development of biotechnological processes aimed at mycoprotein production.

Among the filamentous fungi used for this purpose, *Fusarium venenatum* stands out due to its well-established industrial application in the production of mycoprotein-based foods [23,25]. Its biomass, considered safe for consumption in the United Kingdom since 1984 and subsequently in several other countries, has been reported as a high-value protein source, with protein contents varying according to strain and process conditions and reaching approximately 39% to 76% under optimized conditions [26,27,28,29,30]. This profile is complemented by the presence of essential amino acids and structural cell wall components, such as chitin and glucans, which confer relevant functional properties to the matrix [15,16].

In addition to its composition, studies have associated mycoprotein consumption with positive physiological effects, including increased satiety, modulation of glycemic response, and reductions in blood lipid fractions [31,32,33,34]. The presence of dietary fibers also allows part of this material to be fermented in the gastrointestinal tract, with potential production of short-chain fatty acids, further supporting its interest as a functional ingredient [34,35].

However, most available data refers to reference strains or established industrial lineages. Several non-conventional *F. venenatum* isolates, particularly those obtained from natural environments, still lack studies linking cultivation conditions to biomass yield and final protein content. Since physiological characteristics such as growth, sporulation, biomass accumulation, and protein production are strain-dependent, it is essential to understand how operational variables, such as temperature and agitation, influence the performance of these isolates. Establishing these baseline cultivation parameters is an essential first step for future process optimization and scale-up, supporting the evaluation of the biotechnological potential of new *Fusarium* isolates for mycoprotein production.

Therefore, this study evaluated the growth and sporulation of *F. venenatum* CML3311 on different solid culture media and investigated biomass production under submerged fermentation using different combinations of temperature and agitation. Additionally, this work aimed to determine dry biomass yield and crude protein content under the tested conditions, contributing preliminary data for the characterization of environmental isolates with potential application in the production of protein ingredients.

## 2. Materials and Methods

### 2.1. Fungal Strain

*F. venenatum* CML3311 was obtained from the Coleção Micológica de Lavras (CML), Federal University of Lavras (UFLA), Brazil. The strain was originally isolated in 2015 from *Piper aduncum* collected in São Carlos, São Paulo, Brazil. Prior to the experiments, the strain was reactivated on Yeast Malt Agar (YMA; BD Difco, Sparks, MD, USA; supplied by Interlab, São Paulo, Brazil), composed of yeast extract (3 g/L), malt extract (3 g/L), peptone (5 g/L), dextrose (10 g/L), and agar (20 g/L), with a final pH of 6.2 ± 0.2, and subsequently maintained at 4 °C according to the supplier’s recommendations.

### 2.2. Assessment of Growth in Different Media

To obtain a spore suspension for inoculum preparation, and considering the use of an environmental strain, the growth of *F. venenatum* CML3311 was evaluated on different culture media: YMA; Sabouraud Dextrose Agar (SDA; HiMedia Laboratories, Thane, Maharashtra, India), composed of mycological peptone (10 g/L), dextrose (40 g/L), and agar (15 g/L), with a final pH of 5.6 ± 0.2; Potato Dextrose Agar (PDA; Biolog, Contagem, MG, Brazil), composed of potato infusion equivalent to 200 g/L, dextrose (20 g/L), and agar (15 g/L), with a final pH of 5.6 ± 0.2; and DRBC (Kasvi, São José dos Pinhais, Brazil), composed of peptone (5 g/L), glucose (10 g/L), KH_2_PO_4_ (1 g/L), MgSO_4_ (0.5 g/L), dichloran (0.002 g/L), Rose Bengal (0.025 g/L), chloramphenicol (0.05 g/L), chlortetracycline hydrochloride (0.05 g/L), ZnSO_4_ (0.01 g/L), CuSO_4_ (0.005 g/L), Tergitol (1 mL/L), and agar (12–15 g/L). From a previously grown solid culture, colony fragments measuring approximately 8 mm in diameter were aseptically excised and transferred to plates containing each respective medium. The plates were incubated at 28 °C in a closed incubator without a controlled photoperiod for up to 28 days to determine the suitable period for culture establishment and sporulation. The cultures were briefly exposed to ambient light only during periodic evaluations.

Every 7 days, spore production was monitored by optical microscopy (BA410E; Motic, Xiamen, China) to determine the optimal harvesting time. For this purpose, 5 mL of sterile distilled water was added to the medium surface, and the mycelium was carefully scraped using a microbiological loop to obtain a spore suspension. The suspension was filtered through sterile gauze to remove mycelial fragments, and a 10 µL aliquot was transferred to a Neubauer chamber for spore quantification.

### 2.3. Inoculum Preparation and Submerged Fermentation Conditions

For inoculum preparation, we followed the methodology described by Tong et al. [36], with modifications. Briefly, 50 mL of liquid GYB medium composed of glucose (30 g/L) and yeast extract (8 g/L), with an initial pH of 6.0 ± 0.2, was transferred to a 250 mL Erlenmeyer flask. Subsequently, 1 mL of spore suspension was added (5 × 10^6^ conidia/mL). The culture was incubated at 28 °C for 24 h.

For submerged fermentation, 250 mL Erlenmeyer flasks were prepared containing 100 mL of production medium composed of glucose (60 g/L), yeast extract (0.5 g/L), (NH_4_)_2_SO_4_ (6 g/L), MgSO_4_ (1.5 g/L), KCl (0.7 g/L), Na_2_SO_4_ (0.5 g/L), KH_2_PO_4_ (2 g/L), CaCO_3_ (0.5 g/L), and 10% (*v*/*v*) of the prepared pre-inoculum. The process was conducted for 72 h, with biomass yield monitored every 24 h.

A 3^2^ factorial design, with three levels and two factors, was adopted. The evaluated factors were temperature (26, 28, and 30 °C) and agitation speed (160, 180, and 200 rpm), totaling nine experimental conditions (Table 1). For each condition, nine independent Erlenmeyer flasks were inoculated and organized into three groups corresponding to the sampling times (24, 48, and 72 h). At each time point, three flasks were sacrificed for biomass determination and subsequent analyses, constituting independent biological triplicates.

### 2.4. Biomass Determination

For dry biomass determination, the fermented broth was vacuum-filtered using an autoclavable filtration system equipped with a Whatman GF/C glass microfiber filter (47 mm diameter, cat. no. 1822-047; GE Healthcare Life Sciences, Little Chalfont, Buckinghamshire, UK). The retained biomass was washed with sterile distilled water and dried at 30 °C in a forced-air oven until constant weight, which was typically achieved after approximately 24 h.

### 2.5. Protein Quantification

The crude protein content of the biomass was determined using the Kjeldahl method [37]. Total nitrogen was converted to crude protein using a nitrogen-to-protein conversion factor of 6.25.

### 2.6. Statistical Analysis

Biomass production data were expressed as mean ± standard deviation of three independent biological replicates. For each cultivation temperature (26, 28, and 30 °C), the effects of fermentation time (24, 48, and 72 h) and agitation speed (160, 180, and 200 rpm) on biomass production were evaluated independently using two-way analysis of variance (ANOVA), considering time and agitation speed as fixed factors. When significant differences were detected, Tukey’s multiple comparison test was applied. Statistical significance was established at *p* < 0.05. Crude protein content was determined in duplicate, and results were expressed as mean ± standard deviation. Due to the limited number of replicates, protein data were evaluated descriptively, and no inferential statistical analyses were performed. All statistical analyses and graphical representations were generated using GraphPad Prism version 11.0.2 (GraphPad Software, San Diego, CA, USA).

## 3. Results

### 3.1. Effect of Culture Medium on Fungal Growth

**Figure 1:**
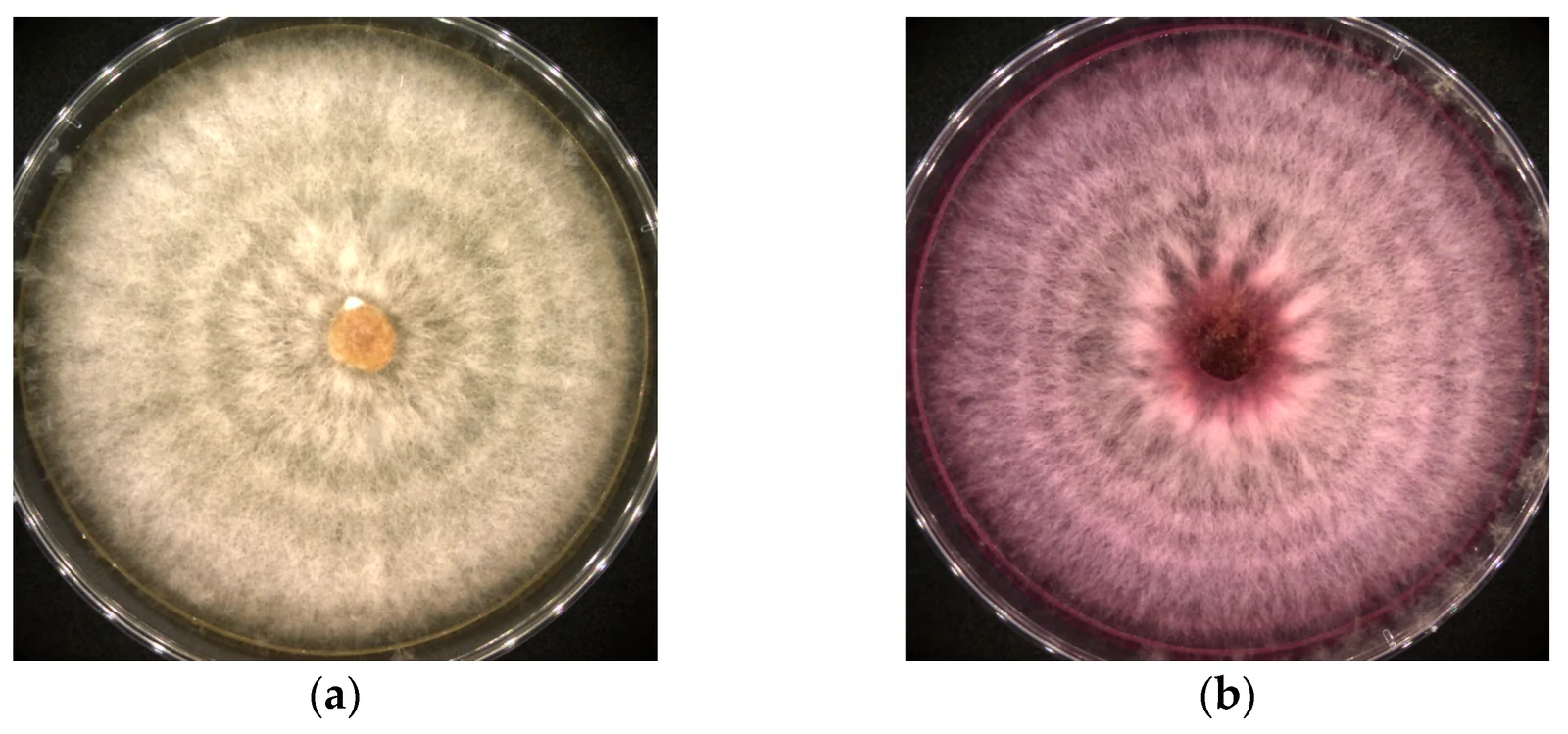

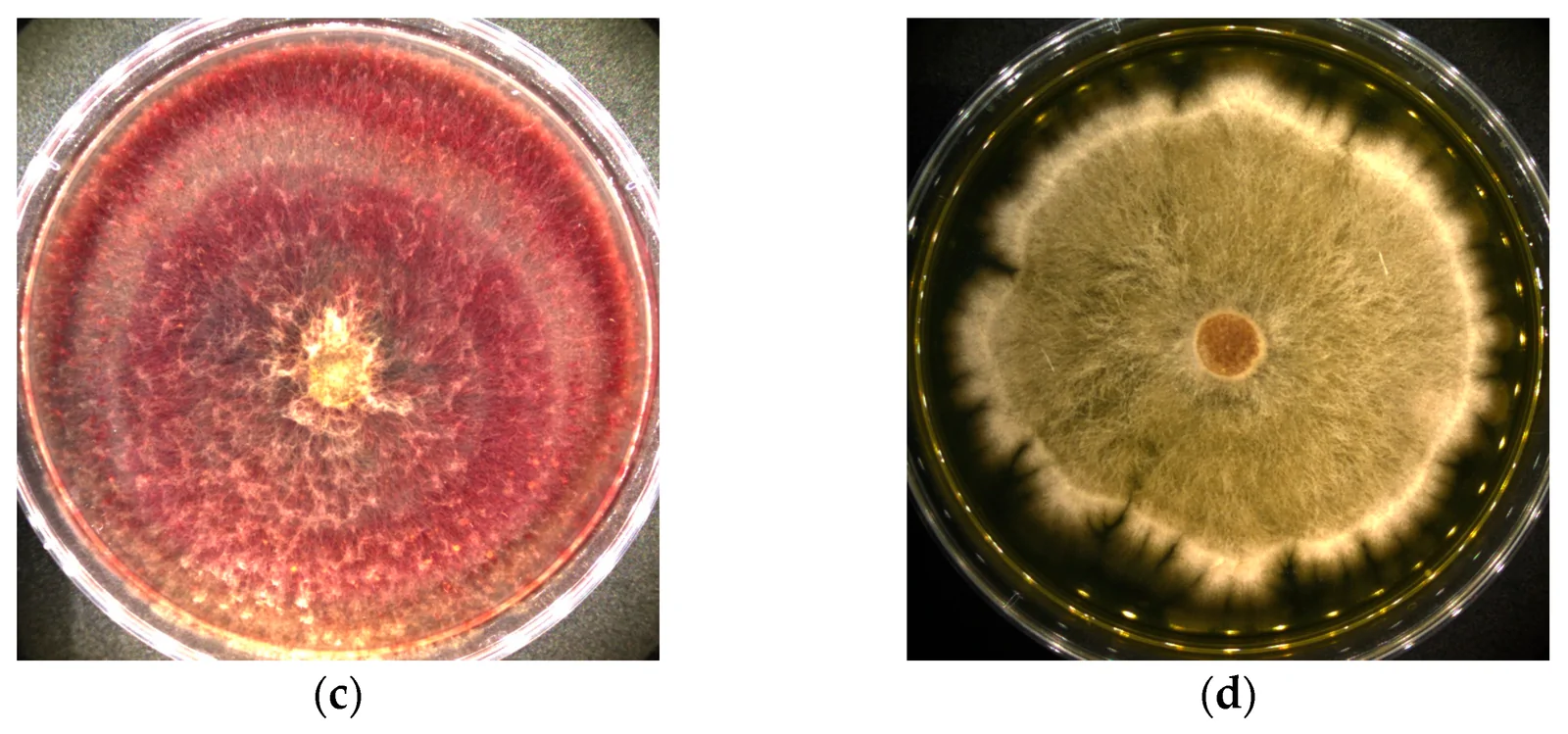
Colony morphology of *Fusarium venenatum* CML3311 grown on different solid culture media after 28 days of incubation at 28 °C. Distinct growth patterns and pigmentation were observed in (**a**) Yeast Malt Agar (YMA); (**b**) Dichloran Rose Bengal Chloramphenicol Agar (DRBC); (**c**) Potato Dextrose Agar (PDA); and (**d**) Sabouraud Dextrose Agar (SDA).

The growth of *F. venenatum* CML3311 varied among the evaluated culture media, with differences observed in macroscopic appearance (Figure 1) and sporulation capacity.

**Figure 2:**
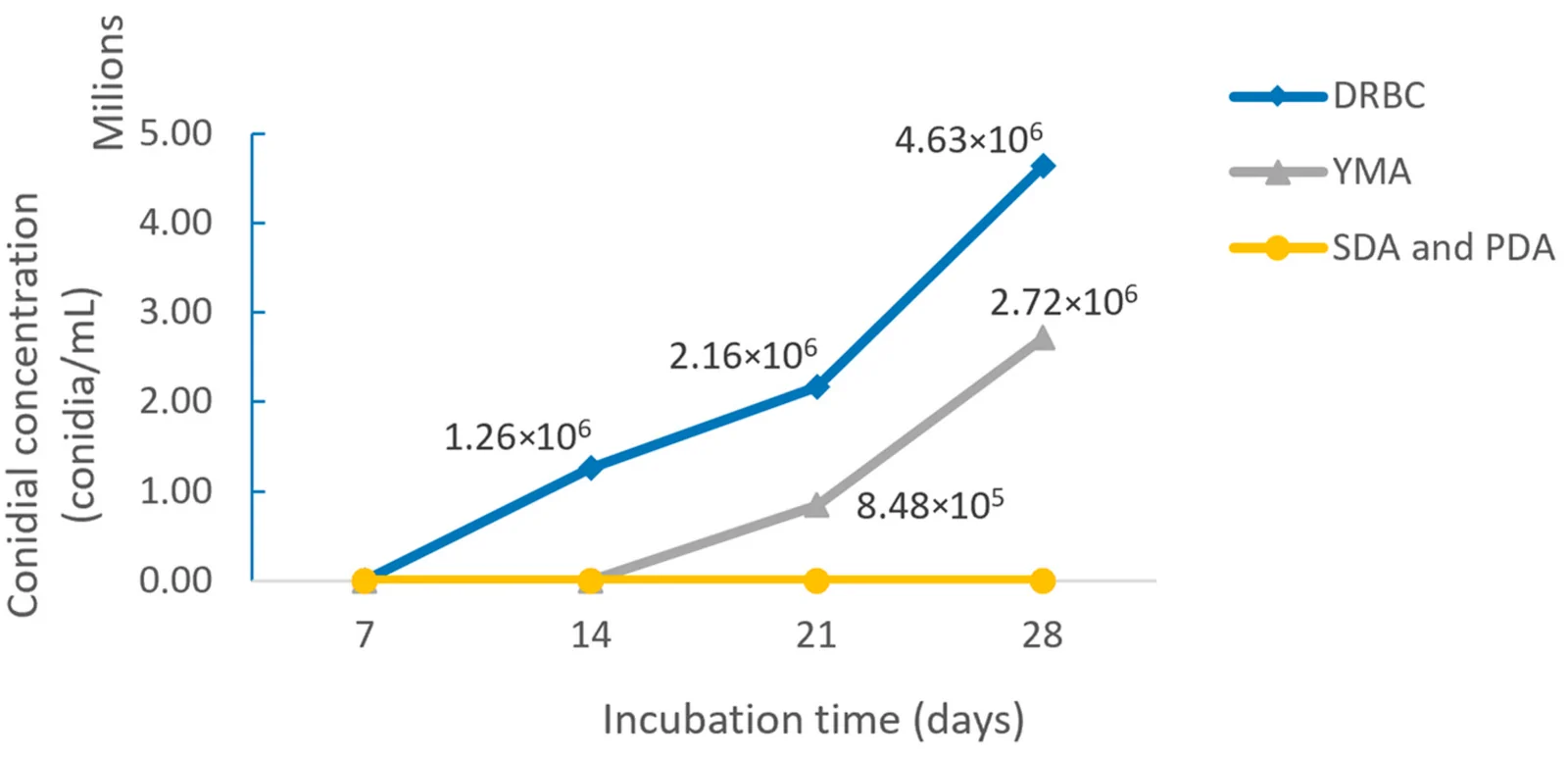
Conidial concentration of *F. venenatum* CML3311 cultured on DRBC, YMA, PDA, and SDA media during 28 days of incubation. Conidial concentration was determined after 7, 14, 21, and 28 days and expressed as ×10^6^ conidia mL^−1^. Values represent the mean of three independent replicates (*n* = 3).

DRBC promoted rapid growth and higher spore production compared with the other media (Figure 1b). Sporulation was observed after 14 days of incubation, reaching 1.26 × 10^6^ conidia/mL, with a progressive increase up to 4.63 × 10^6^ conidia/mL after 28 days (Figure 2). In addition to the higher conidial concentration, colonies grown on DRBC showed homogeneous growth and uniform mycelial distribution across the plate.

On Yeast Malt Agar (YMA), the fungus showed abundant and homogeneous mycelial growth, with a dense cotton-like texture that uniformly covered the agar surface (Figure 1a). Spore production occurred later than on DRBC, being detected only after 21 days of incubation, with a concentration of 8.48 × 10^5^ conidia/mL and reaching 2.72 × 10^6^ conidia/mL after 28 days (Figure 2).

Colonies grown on Potato Dextrose Agar (PDA) showed less dense growth and intense reddish pigmentation (Figure 1c). No conidial production was observed during the evaluated period. A similar result was observed on Sabouraud Dextrose Agar (SDA), which showed sparse growth, more defined colony edges, and absence of sporulation up to 28 days of incubation (Figure 1d).

Considering spore production and colony development, DRBC showed the best performance for preparing the inoculum used in the subsequent fermentation steps.

### 3.2. Biomass Production

**Figure 3:**
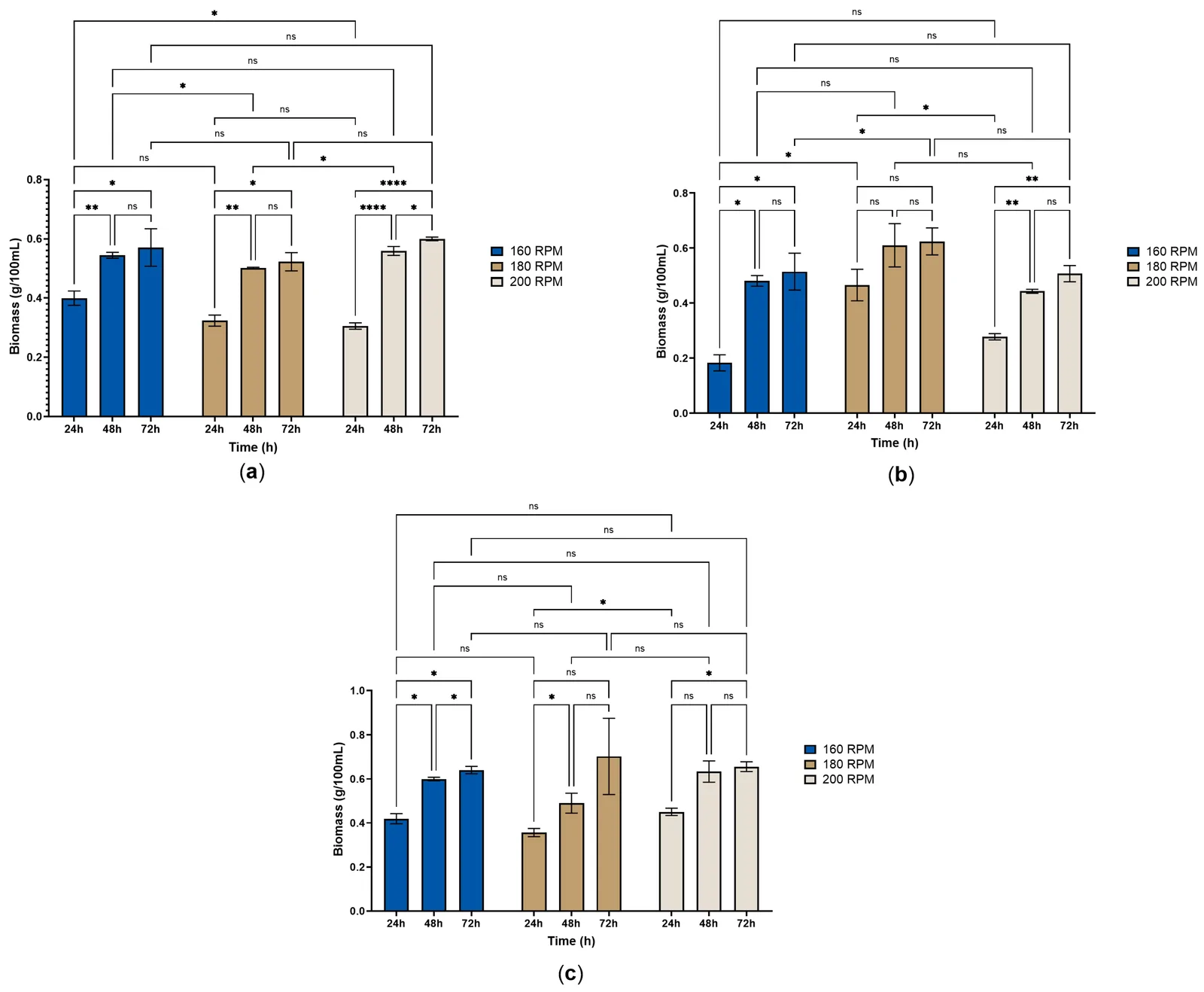
Biomass production by *F. venenatum* CML3311 during submerged fermentation under different agitation speeds and cultivation times at (**a**) 26 °C, (**b**) 28 °C, and (**c**) 30 °C. Biomass concentration was determined after 24, 48, and 72 h of cultivation. Data are presented as mean ± standard deviation of three independent biological replicates (*n* = 3). Statistical comparisons were performed independently for each temperature using two-way ANOVA followed by Tukey’s multiple comparison test. Asterisks indicate significant differences between groups (* *p* < 0.05, ** *p* < 0.01, **** *p* < 0.0001), while ns indicates no significant difference.

Biomass production by *F. venenatum* CML3311 increased throughout the cultivation period under all evaluated conditions, with the most pronounced increase occurring during the first 48 h of fermentation (Figure 3). Overall, biomass values ranged from 0.19 to 0.48 g/100 mL (1.9–4.8 g/L) after 24 h and from 0.52 to 0.65 g/100 mL (5.2–6.5 g/L) after 72 h (Table 2), indicating active fungal growth during the cultivation process.

At 26 °C, agitation speed affected biomass concentration after 24 and 48 h of cultivation. After 24 h, cultures maintained at 160 rpm produced more biomass than those maintained at 200 rpm (*p* < 0.05). After 48 h, the value obtained at 180 rpm was lower than those recorded at 160 and 200 rpm (*p* < 0.05). Biomass increased from 24 to 48 h at all agitation speeds and remained statistically stable between 48 and 72 h (*p* > 0.05), indicating a tendency toward growth stabilization.

At 28 °C, the effect of agitation was more evident during the early stages of cultivation (Figure 3b). After 24 h, biomass production at 180 rpm was significantly higher than that observed at 160 rpm and 200 rpm (*p* < 0.05). However, no significant differences among agitation conditions were detected after 48 h (*p* > 0.05). Similar to the results observed at 26 °C, biomass increased significantly from 24 h to 48 h under 160 rpm and 200 rpm, while no significant changes were observed thereafter (*p* > 0.05).

At 30 °C, agitation speed had a limited influence on biomass accumulation, with significant differences detected only after 24 h, when cultures grown at 200 rpm produced higher biomass than those cultivated at 180 rpm (*p* < 0.05) (Figure 3c). Biomass production increased significantly over time under most agitation conditions, particularly at 160 rpm, where significant increases were observed throughout the entire cultivation period (*p* < 0.05). The highest biomass concentration of the study was obtained after 72 h at 180 rpm (0.65 g/100 mL, equivalent to 6.5 g/L), although no significant differences were observed among agitation conditions at this time point (*p* > 0.05). Overall, cultivation time exerted a stronger influence on biomass accumulation than agitation speed, with biomass production increasing substantially during the first 48 h and approaching a stationary phase thereafter.

### 3.3. Crude Protein Content

**Figure 4:**
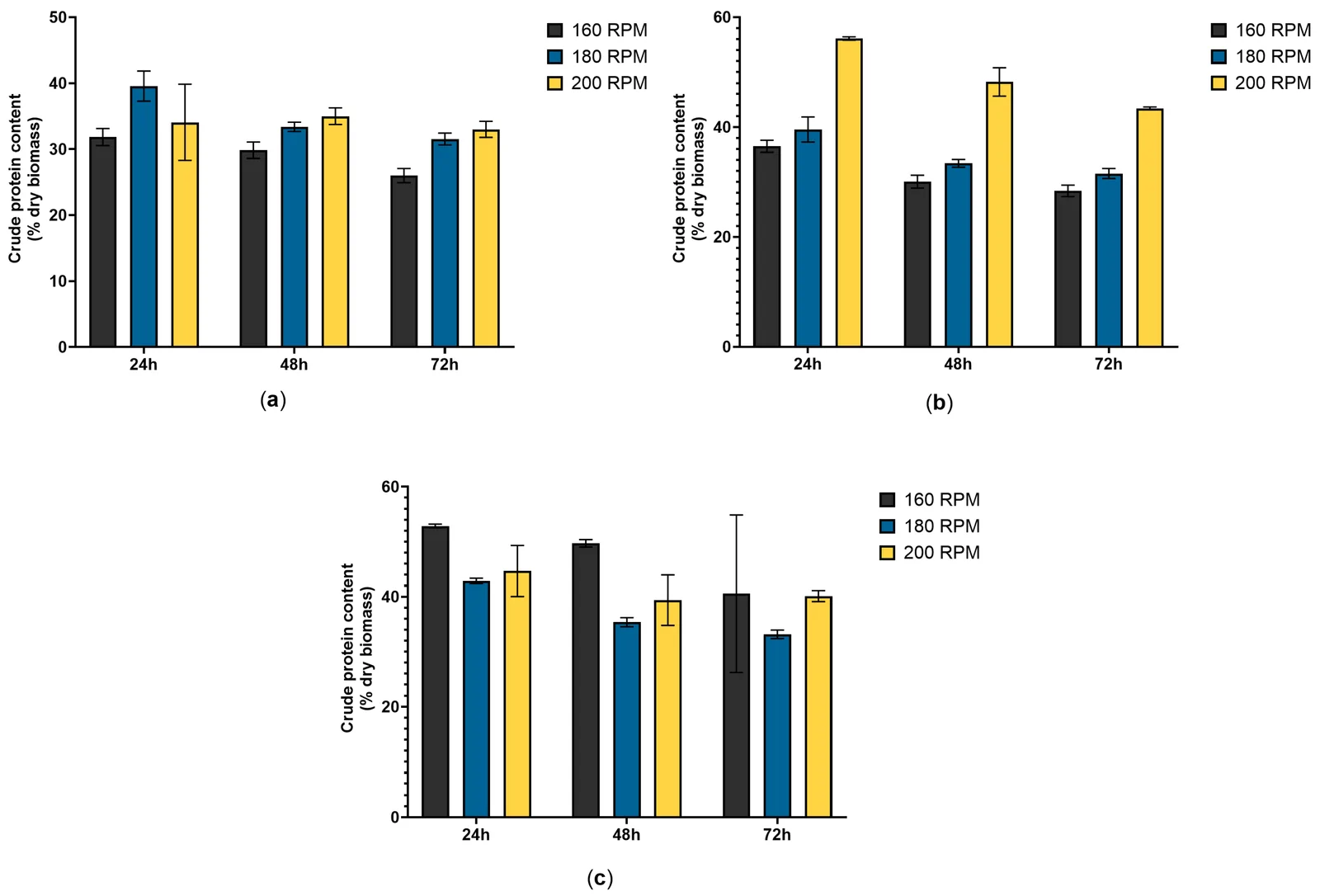
Crude protein content of *F. venenatum* CML3311 biomass produced during submerged fermentation under different agitation speeds and cultivation times at (**a**) 26 °C, (**b**) 28 °C, and (**c**) 30 °C. Protein content was determined after 24, 48, and 72 h of cultivation and expressed as a percentage of dry biomass. Data are presented as mean ± standard deviation of duplicate measurements (*n* = 2).

The crude protein content of *F. venenatum* CML3311 biomass varied according to cultivation conditions and fermentation time (Figure 4). Protein concentrations ranged from 25.45% to 56.12% on a dry weight basis, demonstrating that both temperature and agitation influenced the nutritional composition of the biomass produced.

At 26 °C, crude protein content ranged from 25.45% to 37.20%. In all agitation conditions, crude protein content gradually decreased throughout the cultivation period (Figure 4a). The highest value at this temperature was obtained after 24 h at 200 rpm (37.20%), while the lowest value was observed after 72 h at 160 rpm (25.45%).

At 28 °C, the highest protein contents of the study were recorded (Figure 4b). Biomass produced at 200 rpm exhibited protein concentrations of 56.12%, 48.20%, and 43.41% after 24, 48, and 72 h, respectively. In contrast, cultures grown at 160 rpm and 180 rpm presented lower protein levels, ranging from 26.10% to 35.61%. As observed at 26 °C, protein concentration generally decreased with increasing cultivation time.

At 30 °C, protein contents ranged from 33.16% to 52.86% (Figure 4c). The highest value was obtained after 24 h at 160 rpm (52.86%), followed by a progressive reduction throughout cultivation. Compared with 28 °C, the influence of agitation on protein accumulation was less pronounced, and the differences among agitation conditions were smaller.

Overall, the highest protein concentrations were observed during the first 24 h of cultivation, whereas longer fermentation times were generally associated with lower protein percentages. The most favorable condition for protein accumulation was 28 °C and 200 rpm, which resulted in a maximum crude protein content of 56.12% on a dry weight basis.

## 4. Discussion

The results obtained in this study suggest that the composition of the culture medium influenced the growth and sporulation of *F. venenatum* CML3311. DRBC promoted earlier sporulation, with conidial concentrations reaching 1.26 × 10^6^ conidia/mL after 14 days of incubation. This result is noteworthy because DRBC is a highly selective medium specifically designed for fungal isolation. Its formulation combines Rose Bengal and dichloran, two selective agents that restrict the radial expansion of rapidly growing fungal colonies and have been associated with reduced vegetative growth in several filamentous fungi [38,39], including *Fusarium* species [40].

The earlier sporulation observed on DRBC may be associated with the selective characteristics of this medium. DRBC contains both Rose Bengal and dichloran, which acts synergistically to restrict the radial expansion of rapidly growing fungal colonies while facilitating fungal recovery and isolation without necessarily preventing spore germination [1,2]. By partially limiting vegetative hyphal development, these selective agents may create physiological conditions that favor an earlier transition from vegetative growth to conidiation. In *Fusarium*, sporulation is regulated by multiple environmental cues, including nutrient availability, stress conditions, pH, temperature, oxidative status, and carbon and nitrogen balance [3]. Therefore, the accelerated sporulation observed in the present study likely reflects a strain-specific physiological response of *F. venenatum* CML3311 to the selective properties of DRBC rather than an intrinsic superiority of this medium. This interpretation is consistent with previous reports demonstrating that sporulation responses vary considerably among fungal species and even among isolates of the same species [41,42].

It should also be noted that environmental factors such as light exposure and temperature have been reported to influence colony morphology, zonation, and sporulation in *Fusarium* species [4,5]. In the present study, the cultures were incubated in a closed incubator without a controlled photoperiod and were exposed to ambient light only during periodic observations. Because illumination and short-term temperature fluctuations were not evaluated as experimental variables, the concentric rings observed in the colonies cannot be specifically attributed to these factors. Future studies employing controlled photoperiods and temperature regimes may help clarify their influence on the colony morphology and sporulation of *F. venenatum* CML3311.

In contrast, PDA did not favor vegetative growth or conidial production during the evaluated period, differing from reports described for other *Fusarium* species [40,43]. The intense reddish pigmentation observed on PDA may be related to the activation of secondary metabolism in *Fusarium.* Previous studies have reported that species of this genus can produce red pigments such as fusarubin and related compounds, including naphthoquinones and anthraquinones [44,45]. The synthesis of these compounds is influenced by factors such as nitrogen availability, heat stress, and oxidative stress [46,47,48]. However, because the pigment was not characterized in the present study, its chemical identity could not be determined. Therefore, the observed coloration may be related to secondary metabolite production, potentially favored by nutritional or physiological stress, although this hypothesis still requires analytical confirmation [48,49]. It should be emphasized that the observed pigmentation cannot be considered evidence of mycotoxin production, since pigment biosynthesis and mycotoxin biosynthesis represent distinct secondary metabolic pathways, the activation of which may occur independently [6,34].

During submerged fermentation, biomass production by *F. venenatum* CML3311 increased during the first 48 h under most conditions. Statistical analysis showed that, regardless of temperature, significant differences between 24 h and 48 h were observed in nearly all agitation combinations (*p* < 0.05), indicating that this period concentrated most of the mycelial growth. In contrast, for most evaluated conditions, no significant differences were observed between 48 h and 72 h (*p* > 0.05), indicating stabilization of biomass production after 48 h. The increases recorded after 48 h under specific conditions suggest that the extension of the growth phase may depend on the combined effects of temperature and agitation. The highest biomass concentrations were obtained at 28 °C and 30 °C, reaching approximately 0.64–0.65 g/100 mL, equivalent to 6.4–6.5 g/L, at the end of cultivation. Overall, fermentation time exerted a more consistent influence on biomass accumulation than agitation speed, the effects of which were more evident during the early stages of the process.

From an operational perspective, this behavior suggests that extending cultivation beyond 48 h may not result in substantial biomass gains for strain CML3311, since the increase observed between 48 h and 72 h was not statistically significant under most evaluated conditions. This information is particularly relevant for industrial processes, in which reducing fermentation time may contribute to lowering energy and operational costs.

When expressed in g/L, the highest biomass yields obtained for *F. venenatum* CML3311, ranging from 6.4 to 6.5 g/L, were within the range reported in the literature for the species. Prakash et al. [49] reported a maximum production of 5.40 g/L using Vogel’s mineral medium, while studies employing medium optimization by response surface methodology (RSM) achieved approximately 5.7 g/L after 72 h of cultivation [6,50,51]. The volumes observed in the presente study were also similar to those reported by Tong et al. [52], who obtained 6.49 g/L for a wild-type *F. venenatum* strain cultivated in a 5 L bioreactor. Reported values between 7.2 and 9.5 g/L are generally associated with the use of alternative substrates, advanced optimization of cultivation conditions, or specific fermentation control strategies [29,53]. Biomass yields above 10 g/L have been achieved through genetic modification of the strain and targeted medium supplementation with micronutrients, such as zinc sulfate [52]. Therefore, the yields obtained for strain CML3311 may be considered comparable to those described for *F. venenatum* in the literature, even in the absence of prior medium optimization or specific nutritional supplementation steps.

Regarding the crude protein content of the dry biomass, values ranged from 25.45% to 56.12%, with the highest content observed during the first 24 h of cultivation, particularly under the conditions of 28 °C and 200 rpm. A consistent trend toward reduced protein percentage was observed throughout fermentation, concomitant with increased biomass accumulation. This behavior is consistent with the physiological changes that occur as filamentous fungal cultures mature. During the exponential growth phase, fungal metabolism is primarily directed toward protein biosynthesis to support active hyphal extension and cellular proliferation [9]. As cultivation progresses and growth approaches the stationary phase, the relative proportion of structural cell wall components, such as chitin and β-glucans, increases, while assimilable nitrogen becomes progressively limited and is preferentially allocated to cellular maintenance rather than to the synthesis of new proteins [10]. Consequently, although total biomass continues to increase, the relative crude protein content on a dry weight basis tends to decrease. Similar trends have been reported for filamentous fungi cultivated under submerged fermentation and are considered characteristic of culture maturation and nutrient redistribution [11,12].

The values obtained were within the range described for *F. venenatum* in the literature. Tong et al. [52] reported a protein content of 39.4% for a non-genetically modified strain and 61.9% for a strain subjected to metabolic engineering and zinc sulfate supplementation. Zhou et al. [54] observed 47.84% protein in whole biomass, which increased to 71.22% after high-pressure homogenization. Similarly, Cheriaparambil and Grossmann [28] reported protein contents between 52% and 59% for *F. venenatum* biomass, while Reihani and Khosravi-Darani [29] obtained values up to 76% in bioreactor cultivations using alternative substrates. Thus, the maximum value obtained for strain CML3311 (56.12%) may be considered comparable to those reported for the species under conventional cultivation conditions.

From an industrial perspective, the identification of cultivation conditions capable of producing biomass with high crude protein content during the early stages of fermentation, together with substantial biomass accumulation within 48 h, may represent an important advantage for mycoprotein production. Shorter fermentation cycles can increase reactor turnover, improve volumetric productivity, and reduce energy demand associated with agitation and aeration, which are recognized as major contributors to the operating costs of aerobic fungal fermentations [11,13]. Therefore, process optimization aimed at maximizing productivity over shorter cultivation periods may enhance the economic feasibility of industrial mycoprotein production. Although the present study was conducted at laboratory scale, future investigations should evaluate industrially relevant culture media, fed-batch and continuous cultivation strategies, pilot-scale validation, cultivation temperatures above 30 °C, and comparisons with additional *Fusarium* strains to further optimize biomass productivity and protein yield for industrial applications.

The progressive reduction in protein content observed throughout fermentation is consistent with results described for other fungi cultivated in submerged systems. Bakratsas et al. [55] observed a similar behavior in *Pleurotus ostreatus*, in which protein content reached a maximum during the early stages of cultivation and subsequently decreased, despite continued biomass accumulation. This behavior has been associated with the relative increase in non-protein components, such as structural cell wall polysaccharides and reserve compounds, which represent a greater fraction of dry mass as cultivation progresses. In filamentous fungi, the high proportion of structural carbohydrates may reduce the relative contribution of protein to total biomass, even when protein synthesis remains active [56]. Thus, the results suggest that the first 24–48 h of fermentation represent the most favorable phase for obtaining biomass with higher protein concentration.

Although *F. venenatum* has been commercially exploited for mycoprotein production, the genus *Fusarium* includes species capable of producing several mycotoxins [14]. Previous studies have demonstrated that some *F. venenatum* strains are capable of producing type A trichothecenes under specific cultivation conditions, while genomic analyses identified biosynthetic gene clusters associated with trichothecene production in the species [7,8]. Conversely, studies performed under controlled fermentation conditions with other *F. venenatum* strains have reported the absence of detectable mycotoxins such as deoxynivalenol and zearalenone, indicating that toxin production is highly dependent on the strain and cultivation conditions [6,15]. Therefore, the absence of mycotoxin production cannot be inferred solely from species identification or cultivation performance. Since mycotoxins were not evaluated in the present study, the food safety of biomass produced by strain CML3311 cannot yet be established. Future studies should include targeted analyses of the major *Fusarium* mycotoxins under the selected cultivation conditions before considering this strain for food applications.

Taken together, these findings indicate that both the biomass yield and protein content of the evaluated strain are comparable to those reported for other strains cultivated in modern fermentation systems, even without prior optimization or specific nutritional supplementation. Considering that the present study used glucose as the main carbon source and evaluated only a restricted range of operational conditions, higher yields may potentially be achieved through optimization of medium composition, use of alternative substrates, or supplementation with micronutrients previously described as growth promoters for *F. venenatum*.

## 5. Conclusions

The results demonstrated that culture medium composition influenced the growth and sporulation of *F. venenatum* CML3311. DRBC promoted the highest conidial production and the earliest sporulation, indicating its potential for inoculum preparation. Under submerged fermentation, biomass production increased during the first 48 h of cultivation and tended to stabilize thereafter, with the highest yields observed at 28–30 °C. Crude protein content varied according to fermentation conditions, reaching a maximum value of 56.12% on a dry weight basis, confirming the influence of operational parameters on the quality of the biomass produced.

Overall, the results indicate that *F. venenatum* CML3311 is a promising environmental isolate for mycoprotein production and provides preliminary information for optimizing cultivation conditions aimed at obtaining biomass with a high protein content.

## Figures and Tables

**Table 1:** Experimental design of submerged fermentation parameters.

Nº	Temperature (°C)	Agitation Speed (rpm)
1	26	160
2	26	180
3	26	200
4	28	160
5	28	180
6	28	200
7	30	160
8	30	180
9	30	200

**Table 2:** Biomass production by *F. venenatum* (g/100 mL).

	26 °C			28 °C			30 °C		
	160RPM	180RPM	200RPM	160RPM	180RPM	200RPM	160RPM	180RPM	200RPM
**24 h**	0.41 ± 0.02	0.31 ± 0.02	0.31 ± 0.01	0.19 ± 0.02	0.48 ± 0.05	0.28 ± 0.01	0.41 ± 0.02	0.35 ± 0.02	0.46 ± 0.01
**48 h**	0.55 ± 0.01	0.50 ± 0.00	0.56 ± 0.01	0.49 ± 0.02	0.62 ± 0.06	0.45 ± 0.01	0.60 ± 0.01	0.47 ± 0.04	0.65 ± 0.04
**72 h**	0.60 ± 0.06	0.52 ± 0.03	0.60 ± 0.01	0.54 ± 0.05	0.64 ± 0.04	0.52 ± 0.02	0.64 ± 0.01	0.61 ± 0.14	0.65 ± 0.02

## Data Availability

The original contributions presented in the study are included in the article; further inquiries can be directed to the corresponding author.
